# The Use of UAS to Release the Egg Parasitoid *Trichogramma* spp. (Hymenoptera: Trichogrammatidae) Against an Agricultural and a Forest Pest in Canada

**DOI:** 10.1093/jee/toaa325

**Published:** 2021-02-08

**Authors:** Véronique Martel, Rob C Johns, Laurence Jochems-Tanguay, Frédéric Jean, Alexandre Maltais, Simon Trudeau, Mylène St-Onge, Daniel Cormier, Sandy M Smith, Josée Boisclair

**Affiliations:** 1 Laurentian Forestry Centre, Canadian Forest Service, Natural Resources Canada, Stn. Ste-Foy, Québec, QC, Canada; 2 Atlantic Forestry Centre, Canadian Forest Service, Natural Resources Canada, Fredericton, NB, Canada; 3 Institut de recherche et de développement en agroenvironnement, 335, rang des Vingt-Cinq Est, Saint-Bruno-de-Montarville, QC, Canada; 4 Canopée Dronautique Inc., Montréal, QC, Canada; 5 Anatis Bioprotection, 278 rang Saint-André, Saint-Jacques-le-Mineur, QC, Canada; 6 Faculty of Forestry, University of Toronto, Toronto, ON, Canada

**Keywords:** biological control, drone, agriculture, forestry

## Abstract

The egg parasitoid *Trichogramma* spp. (Hymenoptera: Trichogrammatidae) is a widely used biocontrol agent against lepidopteran pests. Historically, *Trichogramma* were deployed either by plane or by using cardboard cards on which parasitized eggs are glued and manually installed at sites. Plane deployment is costly and card installation is time consuming, but the use of *Trichogramma* has been shown to be efficient against several pests. In 2016 and 2017, a research project investigated the potential use of unmanned aerial system for distributing *Trichogramma* as biocontrol agents against two major pests: an agricultural pest of maize, the European corn borer, *Ostrinia nubilalis* (Hübner) (Lepidoptera: Crambidae), and a forest pest, the eastern spruce budworm, *Choristoneura fumiferana* (Clemens) (Lepidoptera: Tortricidae). Exposure duration of parasitized eggs to field conditions (temperature, predation, etc.) in maize fields influenced the *Trichogramma*’s emergence rate, suggesting that timing of parasitoid releases with their emergence is essential. Although parasitism of naturally occurring eggs in maize fields could not be compared due to the low density of the European corn borer, parasitism of sentinel eggs by *Trichogramma* was more prominent in plots with unmanned aircraft systems (UAS)-releases compared to control plots. For spruce budworm, treatment with *Trichogramma* increased egg parasitism and there was no difference between the deployment by UAS and by Trichocards. We discuss these results in the context of pest biology and management. We also discuss the advantages and shortcomings of both methods and offer insights into where future work might go to further leverage the use of UAS in managing these important pests.

## Graphical Abstract

**Graphical Abstract:**
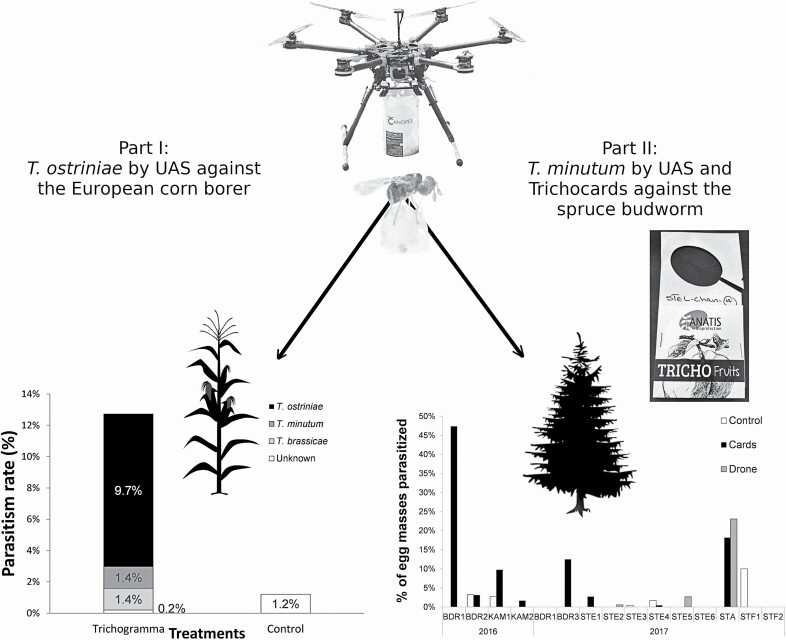

## Résumé

Les parasitoïdes des œufs du genre *Trichogramma* (Hymenoptera: Trichogrammatidae) sont des agents de lutte biologique abondamment utilisés contre les lépidoptères ravageurs. Historiquement, les trichogrammes sont déployés par avion ou par l’installation manuelle de cartes sur lesquelles les œufs parasités sont collés. Bien que l’utilisation d’avion soit coûteuse et l’installation de cartes longue, l’utilisation des trichogrammes s’est avérée efficace contre de nombreux ravageurs. En 2016 et 2017, un projet de recherche a évalué l’utilisation de véhicules aériens sans pilote pour la distribution de trichogrammes contre deux ravageurs majeurs: un ravageur agricole, la pyrale du maïs, *Ostrinia nubilalis* (Hübner) (Lepidoptera: Crambidae), et un ravageur forestier, la tordeuse des bourgeons de l’épinette, *Choristoneura fumiferana* (Clemens) (Lepidoptera: Tortricidae). La durée d’exposition des œufs parasités dans les champs de maïs a influencé le taux d’émergence des trichogrammes, suggérant que la synchronisation de l’épandage des parasitoïdes juste avant leur émergence est essentielle. Bien que le parasitisme des œufs présents naturellement dans les champs de maïs n’ait pas pu être mesuré à cause de la faible densité de pyrale, le parasitisme des œufs sentinelles était plus élevé dans les parcelles traitées avec les drones que dans les témoins. Pour la tordeuse, le traitement avec les trichogrammes a augmenté le parasitisme des œufs et aucune différence n’a été observée entre l’épandage par drone et par tricho-cartes. Ces résultats sont discutés dans le contexte de la biologie et de la gestion des ravageurs. Nous discutons également des avantages et des défis liés aux deux méthodes d’épandage.

Biological control is an essential tool in agricultural and forestry management programs seeking to control pests while avoiding unwanted nontarget impacts (Vincent et al. 2001). While biocontrol agents can be quite effective, they can also be challenging and expensive to deploy, especially in comparison to their chemical insecticide counterparts (Nicot 2011). Such is the case for the often-used biocontrol agent *Trichogramma* spp. (Hymenoptera: Trichogrammatidae), which is a parasitoid that specialize in attacking eggs of a variety of lepidopteran pests (Pinto and Stouthamer 1994). These tiny wasps are the most widely used biocontrol agent in the world (Smith 1996, van Lenteren 2000) because they are easy to mass rear, polyphagous, and specialized to attacking eggs (i.e., the stage preceding most common damaging stage, the caterpillar). In Canada, *Trichogramma* are mainly deployed manually on Trichocards, which are cardboard cards that have *Trichogramma*-parasitized eggs glued to their surface. Trichocard installation is particularly time consuming, leading to recent efforts to find more efficient means of deploying *Trichogramma* under field conditions (Dionne et al. 2018).

In the last 20 yr or so, the use of unmanned aircraft systems (UAS or drones) in different natural resource sectors has increased, including in environmental biology, agriculture, agroforestry, and forestry (reviewed by Paduá et al. 2017, Stone and Mohammed 2017, Nowak et al. 2018, Filho et al. 2020). UAS can improve both the speed and efficacy of pest monitoring, but may also be invaluable to pest management as a less-expensive means of applying insecticides, biocontrol agents, or even sterile insects to disrupt pest reproduction (reviewed by Filho et al. 2020). The United Nations recently highlighted the potential benefits of UAS use in agriculture for monitoring and protecting agriculture, forest, and fishery resources (Sylvester 2018). UAS could improve *Trichogramma* releases for biocontrol by allowing more rapid coverage of larger areas compared with manual distribution and at a much lower cost than helicopter or plane application. Several institutions worldwide have started developing or performing their *Trichogramma* releases by UAS: While it was identified as promising in Brazil for large crops, it was found promising and efficient in China because of the numerous small-sized farms, although the cost has been identified as a limiting factor (Li et al. 2013, Parra 2014, Yang et al. 2018).

In this article, we discuss case studies testing the potential efficacy of using UAS to deploy *Trichogramma* spp. against major pests in agriculture and forestry. The first case study involved the European corn borer, *Ostrinia nubilalis* (Hübner) (Lepidoptera: Crambidae), which is the main pest of processed sweet corn in the province of Québec, Canada (RAP 2018). The European corn borer is difficult to control because after the second instar the larvae bore into the plants’ stems and ears, thus shielding them from insecticides (AAFC 2014). Nevertheless, chemical insecticides are still the main control method used in Québec, which raises concerns for the environment, human health and eventual insecticide resistance. Several field studies with *Trichogramma ostriniae* Pang & Chen suggest that it can limit European corn borer damage by as much as 50% compared to untreated controls (Kuhar et al. 2002, Wright et al. 2002), and thus provides an alternative to conventional insecticides, depending on the pest pressure, and a promising control method for organic production (Gagnon et al. 2017). However, for *T. ostriniae* to be applicable in large-scale maize fields, producers will need more efficient means of deployment than the currently available Trichocards (Boisclair et al. 2011, Dionne 2019).

The second case study involves the eastern spruce budworm, *Choristoneura fumiferana* (Clemens) (Lepidoptera: Tortricidae), a major forest defoliator in eastern Canada. It undergoes outbreak cycles of 30–40 yr, sometimes extending over tens of millions of hectares (Pureswaran et al. 2016). In the province of Québec, Canada, only bioinsecticides are registered for use in forests (MFFP 2000), making Btk (*Bacillus thuringiensis* var. *kurstaki*) the only insecticide currently used for foliage protection for controlling spruce budworm. Although Btk is efficient for foliage protection, some forests or areas are less suitable for its use, including small private woodlots, protected areas, parks, residential areas, camping sites, etc. Although natural enemies such as parasitoids are a major mortality factor in its population dynamics (Eveleigh et al. 2007, reviewed in Pureswaran et al. 2016), they are not sufficient to prevent outbreaks. A large research study during the 1980s showed that proper deployment of the egg parasitoid *T. minutum* Riley—which is naturally present in North American forests—can cause up to 83% of egg parasitism, reducing larval population from 42 to 82%, thus providing significant foliage protection benefits (Smith et al. 1990). Previous studies have shown that both aerial application (with a helicopter) and ground application (with Trichocards) can be effective (Smith et al. 1990).

The specific objectives of the European corn borer case study were to evaluate the survival of parasitized eggs of *T. ostriniae* exposed to environmental conditions on the ground and evaluate the impact of UAS releases on the egg parasitism, presence of larvae and damage on maize farms. The objectives of the spruce budworm case study were to assess the efficacy of *T. minutum* using two different release methods, i.e., by UAS versus the traditional Trichocards. We also described the novel technology developed and used in both case studies, the Entobot (by Canopée Dronautique Inc.). This unit attaches to the bottom of the UAS and carries and regulates the release of *Trichogramma* at predetermined points along a transect. To our knowledge, this is the first published study to report field tests of this approach in Canada.

## Materials and Methods

### Insect Rearing

All *Trichogramma* used in this study were obtained from Anatis Bioprotection (St-Jacques-le-Mineur, QC, Canada). Both species used in this study, *T. ostriniae* Pang & Chen and *T. minutum* were reared on the Mediterranean Flour Moth, *Ephestia kuehniella* Zeller (Lepidoptera: Pyralidae), a common rearing host for *Trichogramma* species. Rearings were maintained at 24°C, 65% RH, 16L:8D. *Trichogramma* spp. were released as parasitized eggs of *E. kuehniella* mixed with fine vermiculite (Holiday, Perlite Canada), a neutral substrate used to give more volume to the *Trichogramma*-parasitized eggs. Mixing and application occurred 24–48 h prior to emergence of parasitoids.

### UAS Releases

#### UAS design

**Fig. 1.**
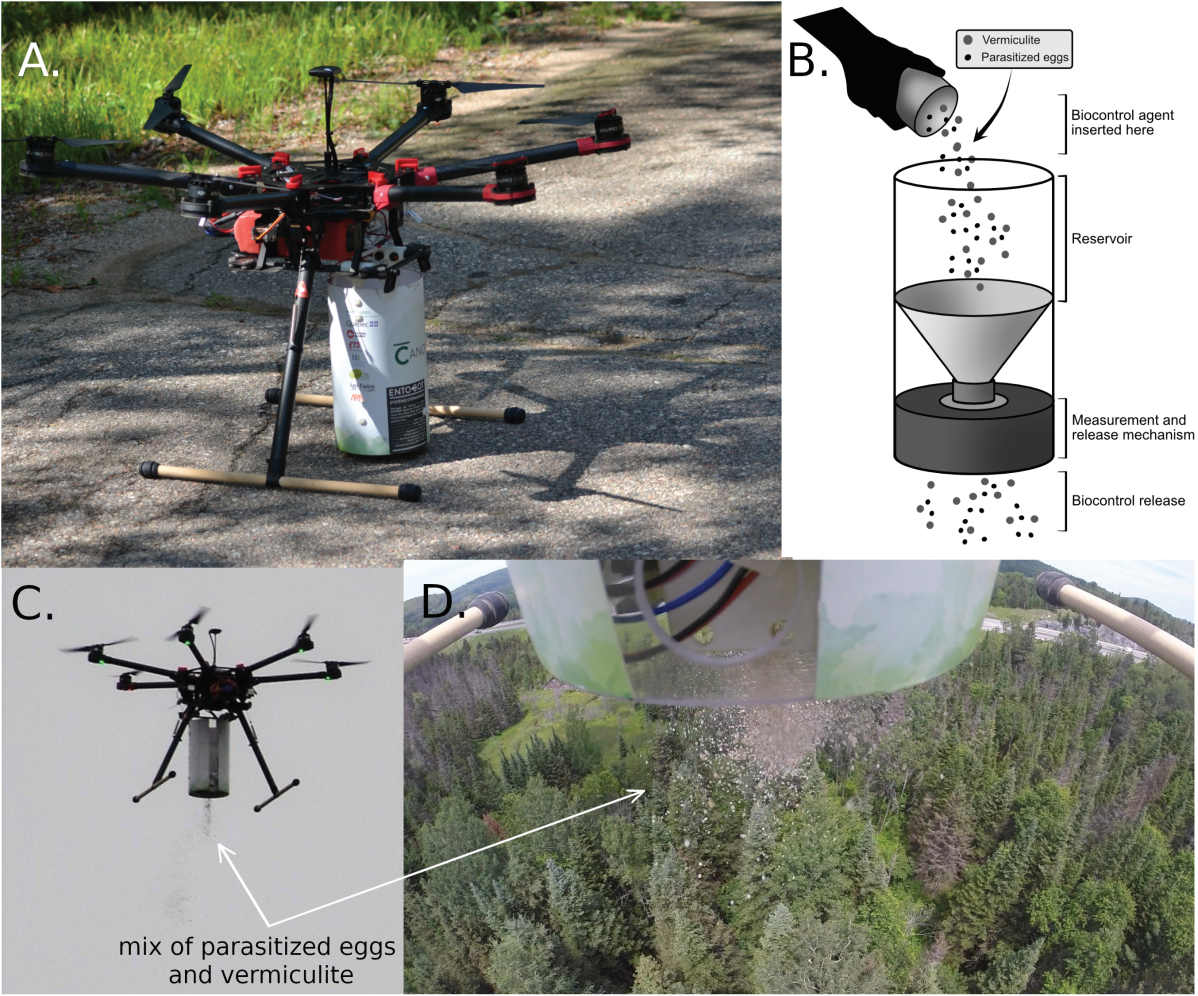
(A) The 900 mm hexacopter aircraft used for the *Trichogramma* releases. (B) Entobot, the spreader designed by Canopée Dronautique Inc. and École de technologie supérieure de Montréal. (C) Picture of the released mix of parasitized eggs and vermiculite taken from the ground and (D) from the UAS during the releases in forests.

The UAS was custom-assembled for the specific task of spreading *Trichogramma*-parasitized eggs following preprogrammed flight paths. The 900 mm hexacopter aircraft was built around the Pixhawk autopilot (3D Robotics, Berkeley, CA). The autopilot controlled all the other UAS components, including the insect spreader (Fig. 1A and B). The Pixhawk is a reliable, open-source autopilot that has been tested and improved upon by a wide base of engineers contributing from academic, governmental and industry sectors. Pixhawk’s open-source nature fostered the development of many different ground control stations (GCS), including Mission Planner (created by Michael Oborne, https://ardupilot.org/planner/) used in this study.

#### Entobot: the beneficial insect spreader

In 2016, Canopée Dronautique Inc. (Montréal, Canada), with the help of École de technologie supérieure in Montréal, designed and manufactured the Entobot, which is the biocontrol agent’s spreader that attaches to the bottom of the UAS. Its main components included a reservoir to hold the parasitized eggs, a measuring mechanism to regulate the release of parasitized eggs, and a gravity-assisted release system at the base of the reservoir (Fig. 1B). The Entobot was designed to have a total takeoff weight of approximately 6.5 kg with its payload of parasitized eggs, well inside the 8.3 kg takeoff weight limit for the aircraft.

For maximum flexibility, the Entobot was designed to offer two release options: 1) the bulk option, with parasitized eggs mixed with vermiculite (Fig. 1C and D); and 2) a small capsule option (single doses of up to 4,000 parasitized eggs). The dual system’s release mechanism consists of adjustable cups in a rotating disk that releases either measured bulk quantities or 1 cm capsules.

#### Trichogramma-parasitized eggs releases

The Entobot released bulk material at specified geographical points along the UAS’s flight path. The flight path consists of GPS coordinates of the optimal path the UAS must follow to cover an area, given the number of releases the spreader must make in that area for a prescribed treatment. The UAS flight path was a two-dimensional scan—a linear back-and-forth path in one axis with equal steps in the other.

The release points were entered in the flight plan using the Mission Planner ground control station. The GPS-guided geographical release allows a more precise distribution of *Trichogramma*-parasitized eggs in a specified area compared to continuous release or time-based point release, countering the UAS’s unavoidable variations in speed due to wind.

During an insect release operation, the UAS flew in ‘automatic’ mode. Before take-off, the flight plan or ‘mission’ (a sequence of geographically based actions that translate to a flight path and release points) was programmed with the ground control station then transferred to the memory of the on-board autopilot. The UAS operator then launched the aircraft in ‘assisted’ or ‘manual’ mode using a remote-control device and proceeded with a series of inflight tests to confirm the system’s airworthiness. If the tests were satisfactory, the operator changed the aircraft’s mode to ‘automatic’. This set off the mission, transferring flight control from the operator to the UAS, which then followed a sequence of GPS coordinates guided by its on-board GPS receiver. Once the mission was completed, the UAS returned to its launch point and landed on its own. If any vermiculite-*Trichogramma* mix remained in the spreader after the release, it was sprinkled on the plot to make sure that the full dose of *Trichogramma* was applied.

#### UAS regulations

The UVA flight regulations in Canada are under the jurisdiction of Transport Canada (Transport Canada 2014). Although regulations have since changed, during the conduct of the study, operators were required to obtain a Special Flight Operations Certificate from Transport Canada for every commercial operation, with delays from several weeks to months. Among the constraints required by the federal regulations, a minimal distance of 30 m had to be maintained between the UAS and any habitation, person or domestic animal, and the UAS had to be kept in the visual line of sight.

### Part I. Agriculture: *Trichogramma ostriniae* Against the European Corn Borer

Two experiments were conducted in the maize production system. The first experiment aimed to evaluate the impact of exposure duration to field conditions (biotic and abiotic) on the ground in the field on the parasitoids’ survival and quality. This experiment simulates what happens to parasitized eggs after UAS release. The second experiment aimed at quantifying the impact of UAS-released *Trichogramma ostriniae* on the European corn borer population and damage.

#### Study sites

**Fig. 2.**
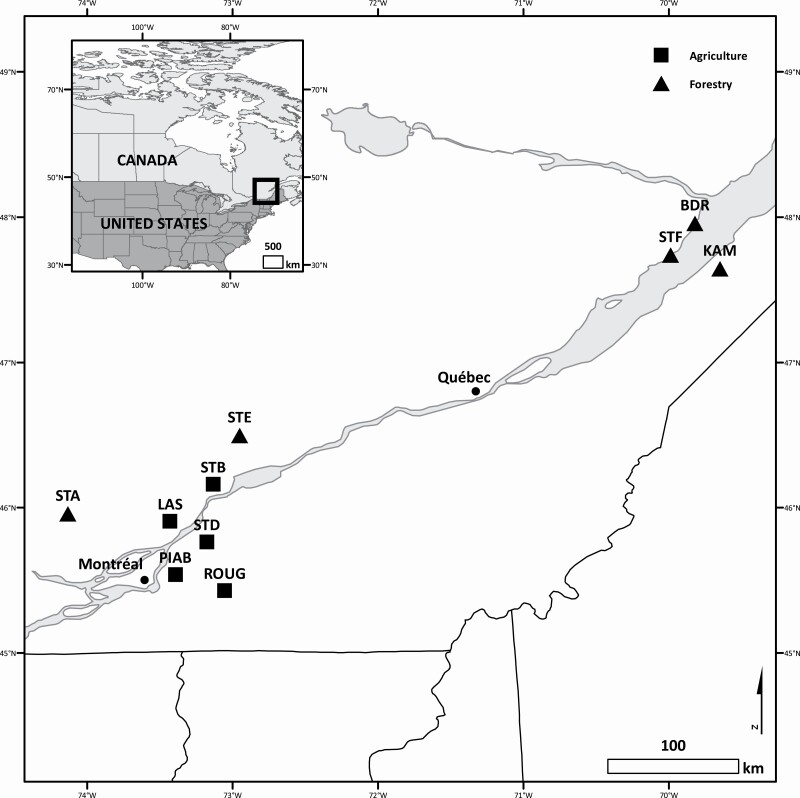
Map showing the different study sites in Québec, Canada.

The experiment on the survival of *Trichogramma* exposed to natural conditions on the ground was conducted in a maize plot planted with the ‘Fastlane’ variety on the Plateforme d’innovation en agriculture biologique (PIAB) managed by the Institut de recherche et de développement en agroenvironnement (IRDA) and located in Saint-Bruno-de-Montarville, Québec, Canada (Fig. 2).

For the UAS releases, four commercial sites were selected, two in the Montérégie region and two in the Lanaudière region in Québec, Canada (Fig. 2). A block of 2–4 ha was selected in each site for UAS releases of *T. ostriniae.* An area of 0.5 ha was selected as far as possible from the treated plot as a control site (Table 1). Insecticides were not applied on any of the plots used in this study. To make sure that the first parasitoid release was carried out at the beginning of moth flight, two pheromone traps (Heliothis type, Distributions Solida, Saint-Ferréol-les-Neiges, QC, Canada) were installed next to each control plot—one for the univoltine strain and one for the bivoltine strain, and were checked twice per week starting on 27 June 2017.

**Table 1.** Detailed information on the agricultural study sites for *T. ostriniae* releases against the European corn borer

Sites	Cultural management	Cultivar	Sowing Date	Area treated with *Trichogramma* (ha)	Distance between treated and control plots (m)	Distance to the nearest site treated with *Trichogramma* in the area (m)
ROUG	Conventional	Slendergen	June 1	4.1	380	80 (from control plot)
STD	Conventional	GH4927 (Syngenta)	July 5	3.0	1000	NA
LAS	Organic	GSS1453 Nontreated	June 12	4.0	120	60 (from treated plot)
STB	Organic	GSS1453 Nontreated	June 13	2.89	80	NA

#### Exposure of parasitized eggs to field conditions

**Fig. 3.**
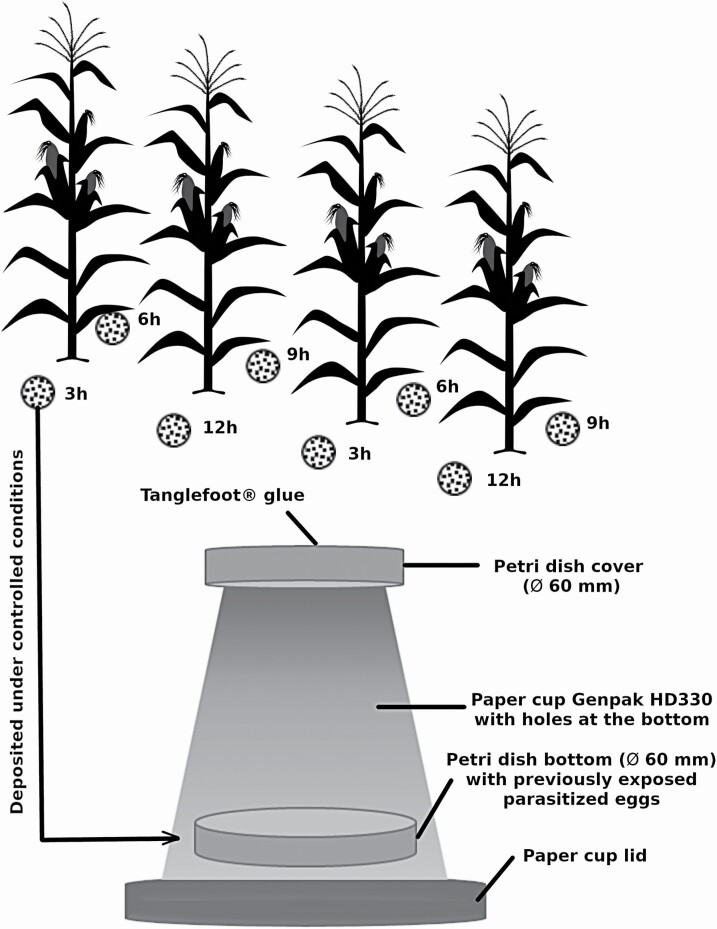
(A) Experimental design showing the Petri dishes containing parasitized eggs in a maize field. (B) Emergence of exposed parasitized eggs in growth chambers.

In order to evaluate the impact of field conditions—both abiotic such as temperature, sun, humidity, and so on and biotic such as predators, pathogens, and so on—parasitized eggs were placed on the ground in maize field for different durations. In 2016, *Ephestia kuehniella* eggs, the rearing host, parasitized by *T. ostriniae*, 24–48h before emergence, were exposed in Petri dishes (Ø 60 mm; 100 eggs/Petri dish, except on July 13 when 50 eggs/Petri dish were exposed) deposited on the ground in a maize field, aligned with a maize plant, but in between rows. Petri dishes were filled with local soil on which carefully selected parasitized eggs were deposited, to simulate a UAS release. These eggs were selected to be in perfect shape to make sure they were viable before exposure. Four different exposure durations were tested: 3, 6, 9, and 12 h. Eggs were exposed starting at 7:00 (EDT) in the morning and removed depending on their treatment. Two controls were also conducted: a positive control where parasitized eggs were deposited on soil without any exposure in the field, and a negative control where parasitized eggs were not exposed in the field and not in the presence of soil. Parasitized eggs were then taken back to the lab and placed in a growth chamber in an emergence trap (Fig. 3) under controlled conditions (23°C, 65% RH, 16L:8D). After 2 wk, rates of emergence, brachyptery (wing malforming or reduction), and sex ratio (proportion of males) were measured for each sample. This experiment was conducted at three different dates: July 13, July 19, and 4 August 2016. For each date, 10 groups of four Petri dishes (corresponding to the four exposure treatments) were exposed randomly in the field.

#### *Trichogramma ostriniae* releases

The second experiment aimed at quantifying the impact of UAS-released *Trichogramma ostriniae* on the European corn borer population and damage. The first release was conducted when the first European corn borer was trapped or when maize reached the six-leaves stage, whichever came first, and was repeated every 7 ± 1 d until 3 wk prior to harvesting, for a total of five releases for all sites, except for the site in Rougemont (ROUG) where six releases were conducted (because the plants were sown earlier; see Table 2). Because of a mechanical problem with the UAS on August 8, the planned releases on that date (LAS and STB) were postponed to 1 wk later. *Trichogramma ostriniae* were released at a rate of ~500k parasitoid/ha (the recommended dose being ~400k/ha, Anatis Bioprotection 2020), with a speed flight around 3–5 m/s at an approximate height of 10 m above ground level, with regularly spaced punctual release points for about 125 release points per ha, for a total of about 11 ml of the vermiculite-*Trichogramma* mix and 4,000 eggs per release point.

**Table 2.** *Trichogramma ostriniae* release dates against the European corn borer in 2017

Sites	Release 1	Release 2	Release 3	Release 4	Release 5	Release 6
ROUG	July 12	July 17	July 23	July 31	Aug. 6	Aug. 14
STD	Aug. 6	Aug. 14	Aug. 21	Aug. 28	Sept. 5	NA
LAS	July 18	July 25	Aug. 1	Aug. 15	Aug. 23	NA
STB	July 18	July 25	Aug. 1	Aug. 15	Aug. 23	NA

At each release date, a subsample of released eggs was sent to the laboratory for rearing to evaluate emergence rate, sex ratio, and brachyptery rate. Eggs were glued on a cardboard strip (0.3 × 1 cm with nontoxic white glue) and placed in a rearing cup (Solo, 1 oz) in a controlled environment for 2 wk (23°C, 65% RH, 16L:8D) and then frozen until observation. The number of males and females of emerged insects were counted, and any adult showing brachyptery was noted. A subsample of 200 eggs from each cardboard was analyzed to count the number of parasitized eggs emerged and unemerged and the number of healthy eggs. Two adults by release date were also barcoded by the Laboratoire d’expertise et de diagnostic en phytoprotection of the Ministère de l’Agriculture, des Pêcheries et de l’Alimentation du Québec (MAPAQ) for identification.

#### Evaluation of European corn borer damage and egg parasitism

Starting when maize plants reached the six-leaves stage, European corn borer survey were conducted every 3–4 d until harvesting on 10 monitoring stations in every plot, each station including five adjacent plants, for a total of 50 plants/plot. During each survey, the number of naturally laid European corn borer egg masses, larvae and plants with European corn borer damage were evaluated. Egg masses were geolocalized so that parasitism could be noted during every subsequent visit.

In addition to European corn borer parasitism, sterilized *E. kuehniella* sentinel eggs were also installed at 10 stations/plot. At each station, one sentinel egg mass was installed underneath a maize leaf at mid-height the morning before the UAS releases. Three to four days later, sentinel eggs were collected and brought back to the lab for rearing (23°C, 65% RH, 16L:8D) for 2 wk, and replaced by new sentinel egg masses, for a total of seven sentinel egg masses/station. Incidence of parasitism was noted when at least one egg was parasitized.

For each sentinel egg mass that was parasitized, two *Trichogramma* adults emerging from the sentinel eggs were sent to the Laboratoire d’expertise et de diagnostic en phytoprotection of the Quebec ministry of agriculture (LEDP-MAPAQ) for molecular identification. The Internal Transcribed Spacer 2 (ITS-2), a highly conserved region in *Trichogramma* species (Stouthamer et al. 1999), was amplified using the following primers: ITS-2F (5′-TGTGAACTGCAGGACACATG-3′) and ITS-2R (5′-GTCTTGCCTGCTCTGCTCTGAG-3′). They were then sequenced by the Plateforme de séquençage et de génotypage des génomes of the Centre Hospitalier Universitaire de Québec – Université Laval (Canada). Sequences obtained were compared with both GenBank (National Center for Biotechnology Information) and reference sequences provided by Dr. Richard Stouthamer (Department of Entomology, University of California).

#### Statistical analyses

All statistical analyses for Part I were conducted on SAS (version 9.4, SAS Institute 2011).

The emergence rates and sex ratio (proportion of males) of parasitized eggs exposed to field conditions were analyzed using a generalized linear mixed model with logit link and binomial distribution, with treatment as a fixed effect and exposure date as a random effect with an overdispersion parameter, with Tukey–Kramer method for posthoc multiple comparisons (Proc GLIMMIX). As no brachyptery was observed, no statistical analysis could be performed on this response variable.

The quality of the parasitoids for each release (emergence rate, sex ratio, and brachyptery rate) was compared among release dates with a linear model with a normal distribution with Tukey–Kramer method for post-hoc multiple comparisons (Proc GLIMMIX).

The number of larvae and the number of plants with European corn borer damage found during inspections were analyzed with a general linear mixed model with a log link and a Poisson distribution with treatment as a fixed effect and site and inspection date as random effects (Proc GLIMMIX). As no European corn borer egg masses were found, no analysis could be performed on their parasitism rates as a response variable.

The parasitism incidence of sentinel eggs by *T. ostriniae* was analyzed using a general linear mixed model with a logit link and a binomial distribution with treatments as fixed effect and sites and dates as random effects (Proc GLIMMIX).

### Part II. Forestry: *Trichogramma minutum* against Eastern Spruce Budworm

#### Study sites

Areas for bioassays were identified based on the budworm population and defoliation levels (MFFP 2015, 2016). We identified four areas where spruce budworm was present with either low defoliation or in a restricted area (Fig. 2). Within each area, we selected one ha blocks each containing paired treated and control plots (30 m × 30 m). It is important to note that in STA, the municipality decided to treat all their forests and wood lots with *Bacillus thuringiensis* var. *kurstaki* (Btk), a bioinsecticide targeting feeding Lepidoptera larvae, to suppress spruce budworm populations. The Btk treatment was applied when spruce budworm larvae were at the fourth instar, end of May, about a month before our experiment.

#### *Trichogramma minutum* releases

To make sure that the first *Trichogramma* release was carried out at the beginning of the moth flight periods, owners or managers of all of our sites were participating in the Budworm Tracker citizen science program trapping spruce budworm moths using pheromone traps (Carleton et al. 2020). The first release was conducted 2–3 d after the first capture and the second release 1 wk after the first one (Table 3). The release rate was 3M/ha (i.e., 1.5M females/ha) for all releases for both years and both methods.

**Table 3.** Dates of the first *Trichogramma minutum* release against the spruce budworm

		2016	2017	
Areas	Blocks	Trichocards	Trichocards	UAS
Kamouraska	KAM1	July 4	NA	NA
	KAM2	July 4	NA	NA
Charlevoix	BDR1	July 11	July 12	NA
	BDR2	July 11	NA	NA
	BDR3	NA	July 12	NA
	STF1	NA	July 12	NA
	STF2	NA	July 12	NA
Mauricie	STE1	NA	July 3	July 3
	STE2	NA	July 3	July 3
	STE3	NA	July 3	July 3
	STE4	NA	July 3	July 3
	STE5	NA	July 3	July 3
	STE6	NA	July 3	July 3
Laurentides	STA	NA	June 30	June 30

**Fig. 4.**
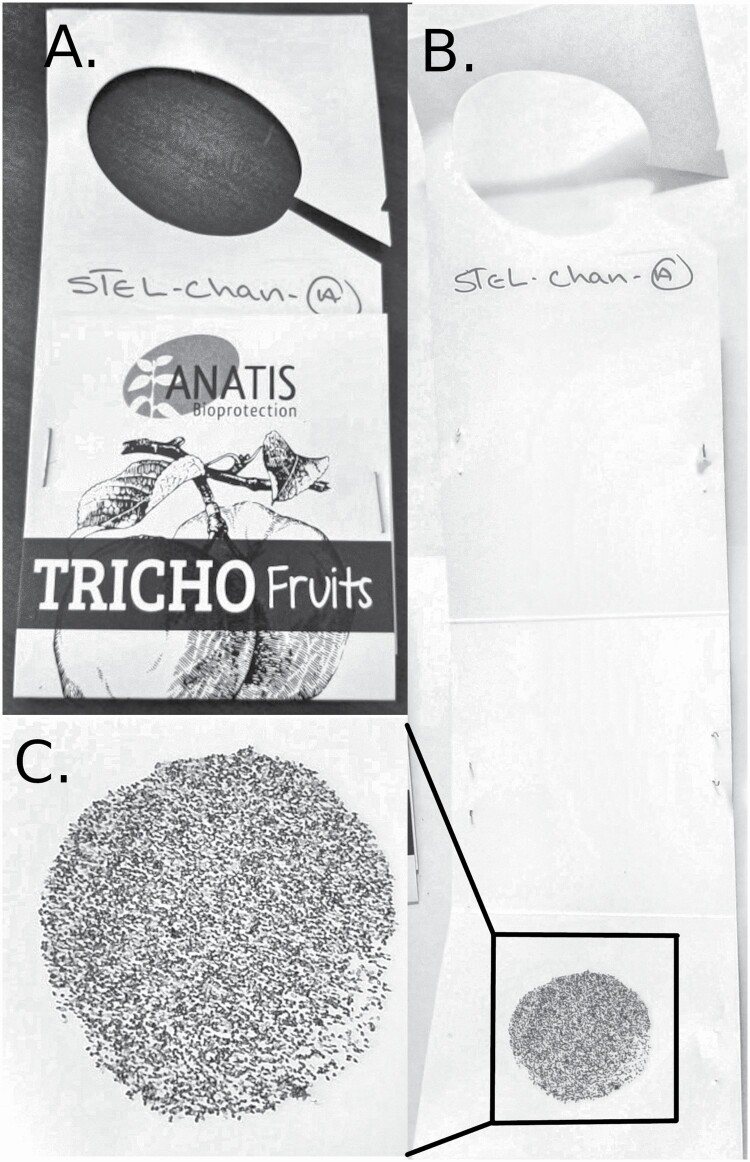
(A) The folded Trichocard on which the parasitized host eggs are glued for releases of *Trichogramma minutum* against the spruce budworm. (B) View of the unfolded Trichocard with a (C) zoom on the glued parasitized host eggs.

Ground releases (using commercial cards provided by Anatis Bioprotection, thereafter called ‘Trichocards’ on which about 6,000 *Ephestia kuehniella* eggs parasitized by *T. minutum* are glued; Fig. 4) were made in each treatment plot to compare both release methods. For each release, 100 Trichocards were installed at eye level on balsam fir or white spruce branches, as evenly as possible within the plot. At the second release, the first Trichocard was left in place to allow for any late emergence and a second Trichocard was installed next to the first one. The Trichocards were removed about a month later, during branch sampling.

UAS releases were done in one plot/block in STE and STA blocks in 2017, on the same day as the Trichocards installation. Flight speed was around 5 m/s at an approximate height of 35 m above the ground (10–20 m above the canopy), with regularly spaced punctual release points for about 750 release points per ha, for a total of about 4,000 parasitized eggs per release point.

#### Egg parasitism

Parasitism rates of natural spruce budworm egg masses was assessed by branch collection: 25 branches of either balsam fir or white spruce (depending on the site composition) of 75 cm, taken at mid-crown level on 25 trees, were collected in each plot, about 10 d after the end of oviposition period as predicted by BioSIM (Régnière et al. 2014). Branches were taken back to the lab and carefully examined for spruce budworm egg masses. Any egg mass found was reared at 20°C, 75% RH, and 16L:8D until parasitoid emergence or larval host hatching. An egg mass was considered as parasitized as soon as one egg was parasitized.

#### Statistical analyses

All statistical analyses for Part II were conducted using the R Software (R Core Team 2017). Parasitism rate were analyzed using a generalized linear mixed model with a binomial distribution with treatment as a fixed effect and block and year as random effects. Post-hoc comparisons among treatments were done using a Tukey multiple comparison procedure approach (glth() function in the multcomp package; Hothorn et al. 2008). Likelihood ratio was calculated using the ‘Anova’ function (package car).

Because *T. minutum* was previously reported to be more effective at low spruce budworm population density (Quayle et al. 2003), the correlation between the parasitism rate and the egg density (number of egg masses/branch) was analyzed with a Pearson correlation tests with log-transformed egg density.

## Results

### Part I. Agriculture: *Trichogramma ostriniae* Against the European Corn Borer

#### Exposure of parasitized eggs to field conditions

**Fig. 5.**
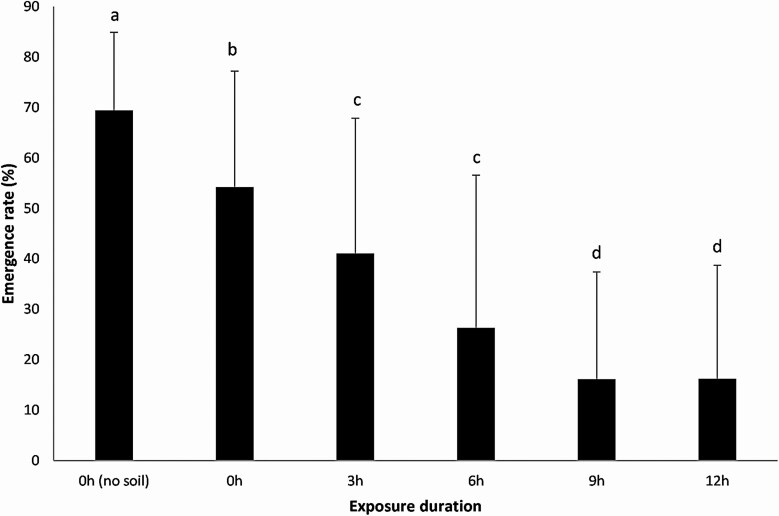
Emergence rate (mean ± SD) of *E. kuehniella* eggs parasitized by *T. ostriniae* deposited on soil in Petri dishes placed on the ground of a maize field exposed for different delays and then reared under controlled conditions. Different letters indicate significant differences (Tukey–Kramer, *P* < 0.05).

Exposure duration in the field had a significant impact on the emergence rate of *T. ostriniae* (*F* = 26.79, df = 5, 75.11, *P* < 0.0001): the emergence rate decreased with the increase length of exposure to the field conditions, and the presence of soil also decreased emergence rate (Fig. 5). There was no impact of the exposure duration in the field on the sex ratio (*F* = 0.35, df = 5, 48.72, *P* = 0.8820), with an overall sex ratio (proportion of males) of 0.39. No brachyptery was observed during this experiment, in any of the treatments for any of the dates.

#### *Trichogramma ostriniae* releases

**Fig. 6.**
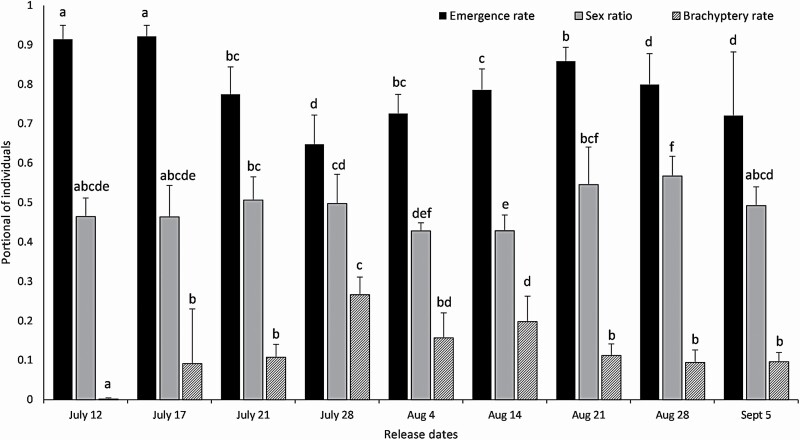
Emergence rate, sex ratio (proportion of males), and brachyptery rates (mean ± SD) of *E. kuehniella* eggs parasitized by *T. ostriniae* subsampled before the UAS-releases at each release date. Different letters indicate significant differences among dates within each variable (Tukey–Kramer, *P* < 0.05).

The sex ratio of released *T. ostriniae* was between 0.37 and 0.66. The quality of the released *T. ostriniae*, evaluated through a subsample from each release date, was significantly different among release dates, for all variable measured: emergence rate (*F* = 34.18, df = 8, 58, *P* < 0.0001), sex ratio (*F* = 3.98, df = 8, 58, *P* = 0.0008), and brachyptery (*F* = 12.16, df = 8, 58, *P* < 0.001; Fig. 6).

#### Evaluation of European corn borer damage and egg parasitism

**Fig. 7.**
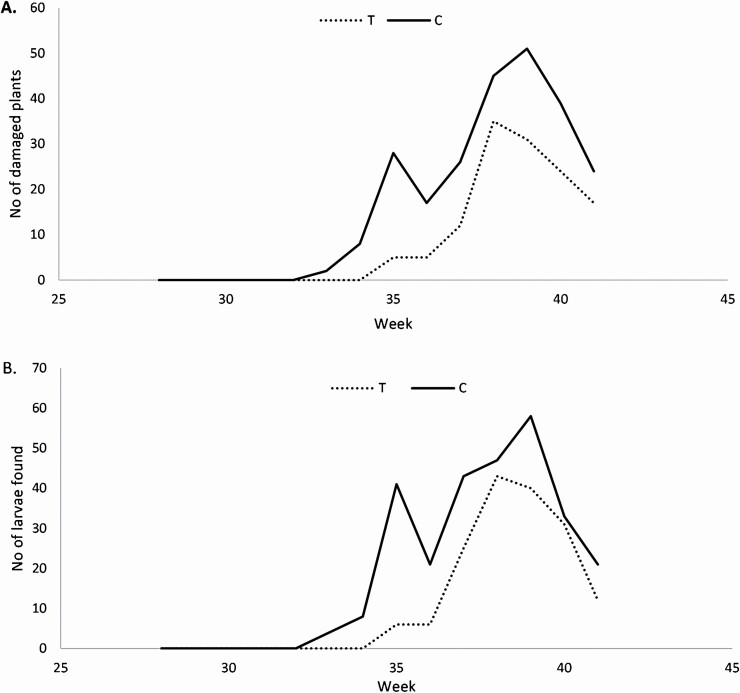
(A) Total number of European corn borer larvae found on maize plants for all plots combined per week for (C) control and (T) treated plots. (B) Total number of maize plants with observed European corn borer damage at each inspection date for all plots combined per week for (C) control and (T) treated plots.

Release of parasitoids had a significant impact on the number of larvae (*F* = 65.19, df = 1, 101.4, *P* < 0.0001) and the number of plants with European corn borer damage found through inspections (*F* = 45.94, df = 1, 146, *P* < 0.0001). In the treated plots, there was an average of 0.073 European corn borer larvae/plot and 0.19% of plants with damage/plot, while for the control plots, there was an average of 0.1248 larvae/plot and 0.46% of plants with damage/plot (Fig. 7). No naturally occurring egg masses were found during inspection on any of the sites. Insects and damage from the true armyworm (*Mythimna unipuncta*), the fall armyworm (*Spodoptera frugiperda*), and the corn earworm (*Helicoverpa zea*) were also found, but not analyzed for this study.

**Fig. 8.**
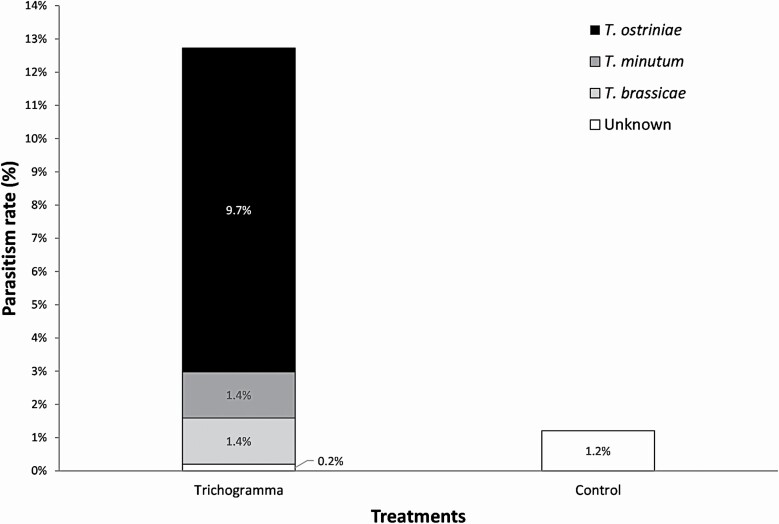
Egg parasitism of sentinel egg masses exposed in maize field during the UAS-releases of *T. ostriniae*.

Sentinel egg masses were parasitized by three *Trichogramma* species according to the molecular results (Fig. 8): the released species, *T. ostriniae*, and two indigenous species, *T. brassicae* and *T. minutum*. Only the parasitism by *T. ostriniae* was analyzed as the objective of the study was to evaluate the efficacy of the releases. Treatment had a significant impact on the parasitism rate of sentinel egg masses (*F* = 12.75, df = 1, 40, *P* = 0.0009), with parasitism being higher in plot where *T. ostriniae* were released.

### Part II. Forestry: *Trichogramma minutum* Against Eastern Spruce Budworm

**Fig. 9.**
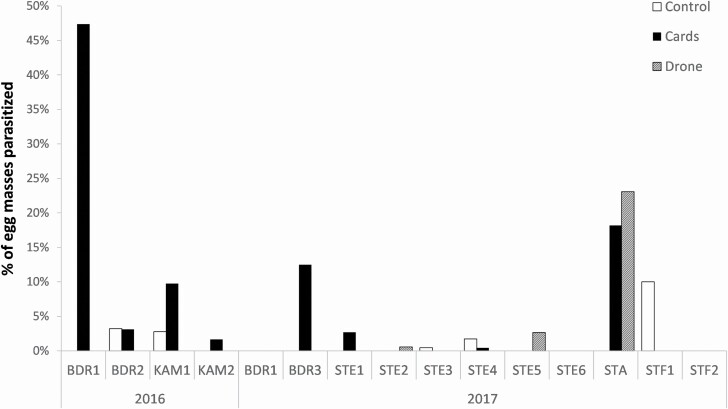
Proportion of spruce budworm egg masses parasitized by *T. minutum* in both 2016 and 2017 for each treatment.

**Fig. 10.**
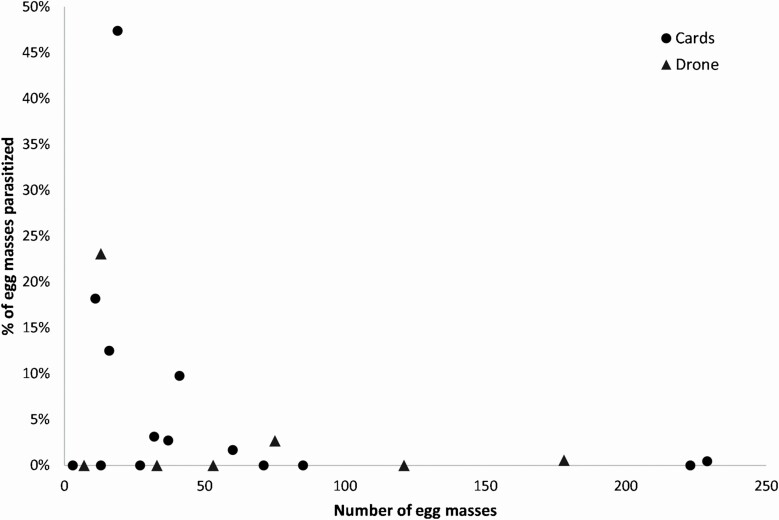
Proportion of spruce budworm egg masses parasitized by *T. minutum* in both 2016 and 2017 in relation to the density of eggs at the site.

Treatment had a significant impact on the spruce budworm egg parasitism rates (LR-χ ^2^ = 9.0125, df = 2, *P* = 0.01104; Fig. 9), with parasitism being higher for both UAS and Trichocards compared to the control (Tukey–Kramer, *P* < 0.05), but not between UAS and Trichocards. There was no significant correlation between the egg parasitism and the egg density for treated sites (*t* = −1.529, df = 20, *P* = 0.0850; Fig. 10).

## Discussion

### Part I. Agriculture: *Trichogramma ostriniae* Against the European Corn Borer

Our results suggested that UAS deployment of *Trichogramma* in European corn borer infested plots could be effective for enhancing parasitism rates during the egg stage. Treated plots had on average fewer European corn borer larvae and fewer plants with European corn borer damage. However, the occurrence of both larvae and damaged plants was generally low, because of the low populations that year, and because the main population was located at one site. Thus, we interpreted results with caution. The objective of this study was not to show the efficacy of the use of *T. ostriniae* against European corn borer in maize, as this has been shown before (Kuhar et al. 2002, Wright et al. 2002, Gagnon et al. 2017), but rather to test UAS as a deployment method. Both the parasitism of sentinel eggs and the difference in number or larvae and damaged plants suggest that using UAS to release *T. ostriniae* could be a reliable method for applying biological control against European corn borer in maize.

Although the European corn borer populations were not high enough to directly measure egg parasitism, the installation of sentinel eggs in our four study sites provided compelling evidence that released *Trichogramma* were able to both locate and attack eggs. Multiple *Trichogramma* species emerged from sentinel eggs, including several indigenous species or species commonly deployed against other agricultural pests, which could be expected considering that *Trichogramma* were commercially released less than 100 m from some of our sites, and can disperse up to 230 m in sweet corn fields (Wright et al. 2001). However, for our released species, *T. ostriniae*, enhanced parasitism rates were observed in treated sites but were totally absent in control plots. This suggests that UAS deployment was effective for distributing the biological control agent.

Although parasitism was detected, parasitism rates of sentinel eggs were below the level considered necessary for suppression of European corn borer populations below relevant economic damage thresholds (Bigler et al. 1994). However, a previous study using *T. ostriniae* against European corn borer showed that the parasitism rates of sentinel *E. kuehniella* eggs were lower than those of natural European corn borer egg masses (Dionne et al. 2018). Another study on the brown marmorated stink bug, *Halyomorpha halys*, also concluded that sentinel eggs underestimate the parasitism rates on the targeted host (Jones et al. 2014). This suggests that the incidence of parasitism on natural European corn borer egg masses could be higher than on the sentinel egg masses from this study.

In our study, parasitized eggs deposited on soil, to mimic what happens after UAS release, and exposed to field conditions (abiotic and biotic) for increasing durations on the ground had lower parasitoid emergence rates, suggesting an impact of either abiotic conditions or predation on survival and emergence. Soil surface temperature can be high and detrimental to *Trichogramma* emergence and survival, especially between corn rows, and it can differ depending on the crop (Orr et al. 1997). Wind could also move the parasitized eggs that are on the ground away from the release site. Predation, especially by ants, can also diminish *Trichogramma*-parasitized eggs released in biological control efforts (Pereira et al. 2004). Thus, releasing parasitized eggs as close to parasitoid emergence as possible is essential when bulk releasing *Trichogramma*, although the risk of parasitoids emerging during transport has to be considered.

Despite some rearing-related variations in life history traits, these variations were not suspected to be sufficiently high to interfere with field parasitism. The sex ratio varied between 0.37 and 0.66, which is standard for mass-reared hymenopteran parasitoids (Clausen 1939, Hamilton 1967). Brachyptery rates were somehow low, the highest rate being slightly below 30%, considering that it can reach level as high as 78.5% in commercial *Trichogramma* (Schmidt et al. 2003). Brachyptery prevents females from flying, but females are still able to search for and parasitize host eggs (Schmidt et al. 2003); its impact is supposed to be variable on the success of biological control (Keller and Lewis 1985 in Schmidt et al. 2003). Emergence rates were high, varying between 60% and 90%.

### Part II. Forestry: *Trichogramma minutum* Against Eastern Spruce Budworm

To our knowledge, this is the first study with the release of biocontrol agents using UAS in forests. Both release methods—Trichocards and UAS—caused an increase in spruce budworm egg parasitism by *T. minutum*. These results are consistent with previous work conducted in Ontario that showed that biological control using *T. minutum* against spruce budworm causes egg parasitism up to 83% (Smith et al. 1990), although the highest parasitism rates in our study was slightly below 50%. However, our release rate of 3M/ha was also lower than the optimal release rate of 10–12M/ha identified by Smith et al. (1990), although the spruce budworm density was also lower in our study. In addition, 2017 was a cold, windy, and rainy summer, and biocontrol agents usually have a lower performance under such conditions (Yu et al. 1984, Smith et al. 1990, Wang et al. 1997, Fournier and Boivin 2000): on cold days, emergence rates are lower and parasitoids fly less and are less active, decreasing the probability of finding and parasitizing the hosts. In addition, in the Mauricie site (blocks STE1 to STE6) where the parasitism rates were generally the lowest, at least two other moth species were observed in abundance during the releases, suggesting additional hosts competing with spruce budworm eggs. In contrast, the second highest parasitism rates were found in the STA site: this was the site where the bioinsecticide Btk was used by the municipality, and the combination of an insecticide decreasing the larval population could have allowed an increase of the parasitism pressure at the egg stage. *Trichogramma* is known to be more effective against hosts at lower population density (Quayle et al. 2003), although we did not see that correlation in our study.

There was no difference in egg parasitism between the plots treated with Trichocards and those treated with the UAS. Smith et al. (1990) also tested ground (by Trichocards and a modified leaf blower) and aerial (by helicopter) releases and had similar results. We thus also conclude that ground or aerial release have similar efficacy. *Trichogramma minutum* is known to search vertically in the tree after emergence (Yu et al. 1984; Smith 1985, 1988), which is not surprising as they naturally parasitize eggs in the upper canopy (Kemp and Simmons 1978, Houseweart et al. 1984), so the localization of the emergence probably has little impact.

The UAS releases were faster and easier to perform than Trichocard installation. Even though we did not specifically measure the time investment required, both teams would start at the same time, but the UAS team was always waiting for the Trichocard team to have finished to move on to the next site. Trichocard installation also required researchers to walk through the forest which, depending on the sites, could be difficult and dangerous depending on the presence of dead trees and branches, roots, or even stinging plants (such as nettle causing contact urticaria) or plant allergens (such as ragweed). Trichocards also needed to be removed after parasitoid emergence, which required an extra site visit.

Although it is not realistic to treat all spruce budworm infested forests using UAS, owing to the massive scale of typical outbreaks, this approach could be advantageous under specific conditions: small areas of private forests, camping sites, parks, protected areas, and so on. Areas of a few hectares where insecticides are either not allowed, or not socially acceptable are suitable sites for *Trichogramma* use.

### Potential and Limits of UAS for *Trichogramma* Releases

From both the agricultural and the forestry parts of this study, UAS seems like an interesting and potentially efficient method for *Trichogramma* releases (Li et al. 2013, Parra 2014, Yang et al. 2018). Even though the Trichocards offer some protection against both abiotic conditions and predation, a tight timing of the UAS bulk release right before parasitoid emergence could offer a good solution, although the costs of parasitoids emerging too early would be even more detrimental. Releasing capsules containing the biocontrol agents could also offer parasitoids a protection. Bulk releases allow the dispersion of parasitoids during release while capsules act more like Trichocards where the parasitoid dispersal comes from punctual points instead of spreading in the field/forest. Because of their preparation, Trichocards and capsules also require more work, which of course adds to costs. However, preparing capsules is faster than preparing Trichocards, given that the proper installations are available (Anatis Bioprotection, personal communication).

Of course, UAS also carry operation costs, that may vary based on whether they are done by producers, forest managers, or by service providers. However, it is reasonable to expect a decline in the operating costs of UAS because of their increasing usefulness in many different sectors. An increase in the number of UAS operators should also encourage competition and lower costs.

While Trichocards can be installed under any weather conditions, UAS have some constraints regarding mainly winds, but also rain depending on the model, and their flight duration is limited by the battery autonomy. However, next-generation UAS have higher flight autonomy and more resilience in high winds. It is possible to adjust UAS flight missions used for recurrent treatments in one location to compensate for stable, stronger than average winds by moving the area to cover in Mission Planner ground control station.

The regulations surrounding the use of UAS can also have an impact on its use for biological control applications. Since the study was conducted, the process received gradual improvements in Canada, until June 2019, when the Canadian government completely overhauled the system (Transport Canada 2019). Now, any UAS operator who wants to use a craft weighing between 250 g and 25 kg can do so freely after obtaining an operator certificate (for basic or advanced operations) and registering their aircraft. The new rules removed the administrative bottlenecks that hindered the commercial use of UAS. It is worth noting that passing the exam to obtain either one of the new operator certificates require dozens of hours of preparation. Getting some training in a private pilot schools is highly recommended. For this reason, producers and forest managers are likely to entrust the service to specialized UAS operators rather than purchasing and self-operating the UAS. The new regulations still impose constraints concerning the visual line of sight, the minimal distance of 30 m maintained between the UAS and any habitation, person or domestic animal, and a minimal distance from airports. These are not insurmountable though, as requests can be coordinated with NAV CANADA, Canada’s civil air navigation services provider.

UAS also brings the benefit of being potentially coupled with imagery that could detect the presence of the pest, and more precisely release biocontrol agents only on the specific part of the field/forests that need them, avoiding non-host or healthy plants (reviewed by Filho et al. 2020). Of course, a lot of development will likely occur and improve the UAS release technique. Among the questions that could be asked and tested: Should the system release parasitized eggs only, a mix of parasitized eggs with a neutral substrate, or capsules with parasitized eggs inside them? Should the system release material continuously along the UAS flight path, at a timed frequency or at predetermined geographical points? Additional studies in different systems will be needed to answer these questions.
